# The histone methyltransferase EZH2 as a druggable target in SHH medulloblastoma cancer stem cells

**DOI:** 10.18632/oncotarget.19782

**Published:** 2017-08-02

**Authors:** Evelina Miele, Sergio Valente, Vincenzo Alfano, Marianna Silvano, Paolo Mellini, Diana Borovika, Biagina Marrocco, Agnese Po, Zein Mersini Besharat, Giuseppina Catanzaro, Giuseppe Battaglia, Luana Abballe, Clemens Zwergel, Giulia Stazi, Ciro Milite, Sabrina Castellano, Marco Tafani, Peteris Trapencieris, Antonello Mai, Elisabetta Ferretti

**Affiliations:** ^1^ Center for Life NanoScience@Sapienza, Istituto Italiano di Tecnologia, Rome 00161, Italy; ^2^ Department of Chemistry and Technologies of Drugs, Sapienza University of Rome, Rome 00185, Italy; ^3^ Department of Experimental Medicine, Sapienza University of Rome, Rome 00161, Italy; ^4^ Latvian Institute of Organic Synthesis, Riga LV-1006, Latvia; ^5^ Department of Molecular Medicine, Sapienza University of Rome, Rome 00161, Italy; ^6^ Neuromed Institute, Località Camerelle, Pozzilli 86077, Italy; ^7^ Department of Pharmacy, University of Salerno, Fisciano 84084, Italy; ^8^ Department of Medicine and Surgery, University of Salerno, Baronissi 84084, Italy; ^9^ Pasteur Institute, Cenci-Bolognetti Foundation, Sapienza University of Rome, Rome 00185, Italy; ^10^ Current address: Department of Hematology/Oncology and Stem Cell Transplantation, Bambino Gesù, 28 Children's Hospital, IRCCS, Rome 00165, Italy

**Keywords:** histone methyltransferase, EZH2 inhibitors, hedgehog pathway, medulloblastoma stem-like cells, self-renewal

## Abstract

The histone methyltransferase EZH2 plays a role in maintenance of the stem component of cancer, and its overexpression and/or mutation typically drives tumor aggressiveness, drug resistance and patients’ poor prognosis. In this study, we use mouse and human medulloblastoma stem-like cells belonging to the Sonic Hedgehog subgroup (SHH MB-SLCs) and demonstrate that genetic suppression of EZH2 reduces the level of its histone mark H3K27me3 and lowers proliferation and self-renewal. We designed an EZH2 inhibitor (EZH2i) as a simplified analog of EPZ005687 and GSK2816126, MC3629, and we tested its biological activity in SHH MB-SLCs. Pharmacological inhibition of EZH2 impairs SHH MB cells proliferation and self-renewal, and induces apoptosis *in vitro*. Finally, we generated xenograft MB-SLCs orthotopic tumors in nude mice to test MC3629 *in vivo*. In treated mice, we observed impairment of tumor growth, together with induction of apoptosis and reduction of proliferation and stemness.

Overall, these findings describe EZH2 as a druggable target in MB and provide insight into the biological activity of MC3629 as an EZH2i.

## INTRODUCTION

The histone methyltransferase (HMT) Enhancer of Zeste Homolog 2 (EZH2) is the catalytic subunit of the polycomb repressive complex 2 (PRC2) [1], which is responsible for methylation of histone H3 on lysine 27 up to its trimethylated form (H3K27me3) on the promoter region of target genes. EZH2/PRC2 induces an epigenetic change that regulates transcriptional silencing [2–4] and plays a pivotal role in different biological processes including differentiation, maintenance of cell identity and proliferation [5]. EZH2 overexpression and mutations occur in several malignancies [6] and are usually associated with aggressive tumors, drug resistance and poor prognosis [7–9].

As far as brain cancers, EZH2 expression increases with tumor grade in adult and pediatric tumors and it is a poor prognostic factor [10]. Medulloblastoma (MB) is the most common malignant pediatric brain tumor [11] and is among those tumors that overexpress EZH2 [12, 13]. EZH2 genetic or pharmacological targeting has been shown to impair MB cells growth [12].

Interestingly, EZH2 is known to sustain self-renewal of cancer stem-like cells (SLCs) [14, 15]. In the hierarchical organization of tumor-cell populations envisioned by the cancer stem cell hypothesis, initiation, maintenance, and advancement of tumor growth are considered mainly properties of the SLCs compartment. Thus, EZH2-targeting drugs have become attractive weapons in cancer therapy [16] and research effort on developing enzymatic inhibitors is fueled [17]. Nevertheless, *in vivo* use of 3-deazaneplanocin A (DZNep) [18], a *S*-adenosyl-L-homocysteine (SAH) hydrolase inhibitor strongly effective against EZH2 *in vitro*, has been stopped because of toxicity in mice [19], and some catalytic *S*-adenosyl-L-methionine (SAM)-competitive EZH2 inhibitors (EZH2i) available for clinical use at the beginning of our study, such as EPZ005687 [20] and GSK2816126 [21], have a limitation for their use due to the poor bio-distribution *in vivo* and scarce ability to cross the blood brain barrier (BBB) [22, 23].

In this study we assessed the EZH2 expression and role in the SLCs derived from MB belonging to the Sonic Hedgehog (SHH) subgroup, namely SHH MB-SLCs [11]. Having established the key function of EZH2 in this cellular context, we tested a simplified analog of EPZ005687 and GSK2816126 prepared by us, the *N*-((4,6-dimethyl-2-oxo-1,2-dihydropyridin-3-yl)methyl)-5-methyl-1-phenyl-1*H*-pyrazole-4-carboxamide MC3629, to determine its ability to target EZH2 and to impair SHH MB-SLCs *in vitro* and *in vivo*.

We show that either genetic or pharmacologic inhibition of EZH2 suppresses MB-SLCs, and in detail that MC3629 is able to impair MB proliferation/growth including its SLC compartment.

## RESULTS

### Genetic inhibition of EZH2 impairs SHH MB cells proliferation and self-renewal

We first assessed EZH2 protein levels in a series of MB cellular models including SHH MB-SLCs from both mouse models (Ptc1^−/+^ mice) (mMB SLCs_1-3_) and human primary tumors (hMB SLCs_4-6_) that we had previously characterized [24].

**Figure 1:**
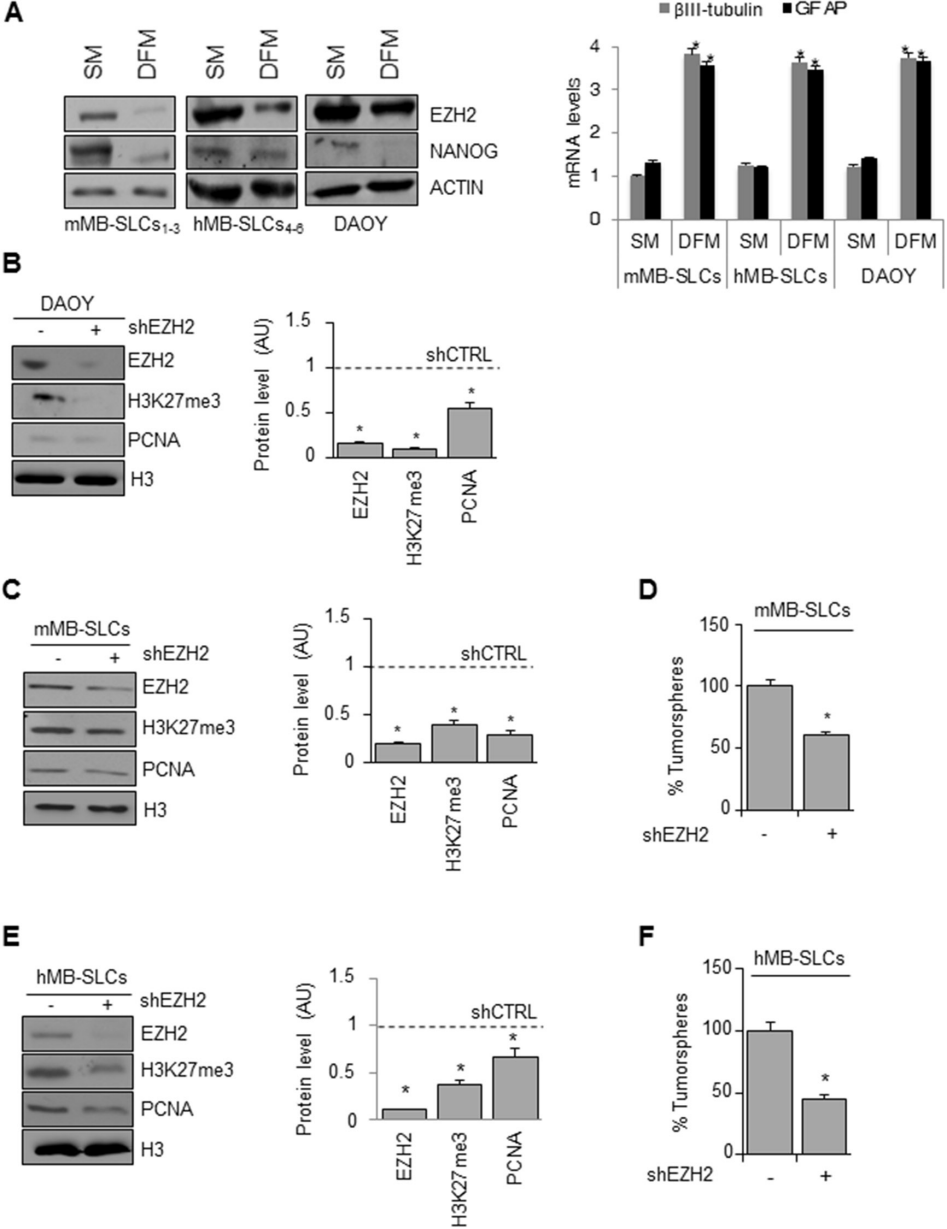
Genetic EZH2 knockdown impairs MB cell proliferation and self-renewal **(A)** Left: representative Western Blot for EZH2 and NANOG protein levels in mMB-SLCs, hMB-SLCs and DAOY cells grown in SM and DFM. ACTIN was used as loading control. Right: mRNA levels of βIII-tubulin and GFAP of all the three models in SM and DFM. *p<0.05. **(B-C-E)** Left: representative Western Blot showing protein levels of EZH2, H3K27me3 and PCNA before and after shEZH2 in DAOY cells (B), mMB-SLCs (C) and hMB-SLCs (E). Dashed bars represent shRNA-non-target control (shCTRL). H3: loading control. Right: densitometric analysis of protein levels from three independent experiments. *p<0.05. **(D-F)** Self-renewal ability (percentage of tumorspheres) of mMB-SLCs and hMB-SLCs after shEZH2 or shCTRL. *p<0.05.

We found high expression of EZH2 in both mouse and human MB-SLCs that grow as oncospheres in stem cell-medium (SM) and lower levels in cells grown in FBS-supplemented medium (DFM) (Figure 1A, left). In these conditions, MB-SLCs acquired a “differentiated” phenotype with adherent morphology, expressing both neuronal and glial lineage markers (βIII-tubulin and GFAP, respectively) and reducing the expression of the stemness marker NANOG (Figure 1A). We also evaluated EZH2 levels in MB DAOY cells [25]. DAOY cells cultured for three days in SM, DAOY-SLCs, exhibited higher protein levels of EZH2 and NANOG, whereas they exhibited lower levels of βIII-tubulin and GFAP, when compared to DFM DAOY culture (Figure 1A).

First we tested three different lentiviral-mediated short hairpin silencing for EZH2 in both mouse and human MB-SLCs (Supplementary Figure 1A-1B), and then we used the most effective ones (**a** and **d** clones, respectively) for further experiments in the three cellular models described above. ShEZH2-transduced DAOY cells showed decreased levels of H3K27me3 together with impairment of cell proliferation (Figure 1B, 1C and 1E). Moreover, in shEZH2-transduced MB-SLCs we observed reduced self-renewal capacity (Figure 1D and 1F).

To further validate our results, we sorted hMB-SLCs for the CD133 stemness marker (Supplementary Figure 1C) [24] obtaining positive (CD133+) and negative (CD133-) hMB-SLCs fractions, that were transduced using lentiviral shEZH2. ShEZH2 impaired cell viability in CD133+ hMB-SLCs more significantly than in CD133- hMB-SLCs (Supplementary Figure 1D).

Our results show that EZH2 plays a critical role in the maintenance of MB cells including SLCs.

### Pharmacologic inhibition of EZH2 with a pyrazole-based EZH2 inhibitor impairs SHH MB-SLCs cell viability

**Figure 2:**
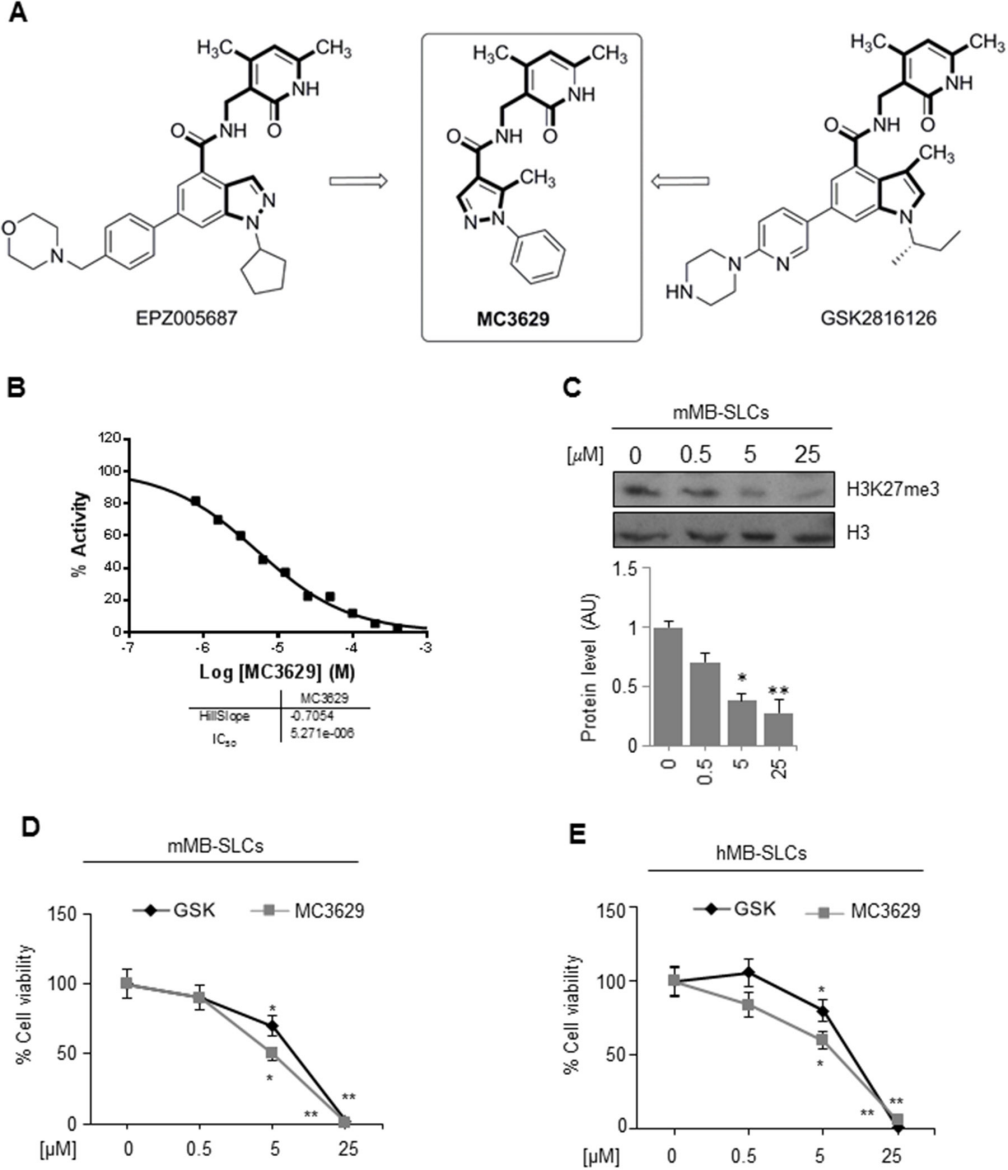
The EZH2 inhibitor MC3629 impairs SHH MB-SLCs viability **(A)** Design of MC3629 starting from modification of the EPZ005687 and GSK2816126 structures. **(B)** EZH2/PRC2 assay in 10-dose IC_50_ mode with 2-fold serial dilution starting from 400 μM solution. Histone H3/H4 tetramer was used as the substrate. **(C)** Representative Western Blot (up) and densitometric analysis (down) of H3K27me3 protein levels in mMB SLCs treated for 48 h with 0.5, 5 and 25 μM of MC3629. *p<0.05, **p<0.01. **(D-E)** Evaluation of cell viability, measured with MTS assay, of mMB-SLCs (D) and hMB-SLCs (E) treated with increasing concentrations of MC3629 and GSK2816126 at 48 h. p value versus untreated cells: *p<0.05, **p<0.01.

EZH2 is a druggable target in different cancers [6], and commercial catalytic EZH2i, such as EPZ005687 and GSK2816126 (Figure 2A), are used in lymphoma with EZH2-activating mutations and also in solid tumors [20, 21, 26]. Nevertheless, there are reports regarding limitation to EZH2i use *in vivo* due to poor bio-distribution and scarce ability to cross the BBB [22, 23].

Following our researches on design, synthesis and biological evaluation of histone lysine methyltransferase inhibitors [27–32], we developed the pyrazole-based small molecule MC3629 as a simplified analog of EPZ005687 and GSK2816126. To design MC3629, we modified the structure of these two known EZH2i removing the benzene ring from the indazole or indole nucleus, respectively, to obtain a monocyclic nitrogen-containing heteroaromatic ring (the pyrazole ring) linked through a carboxamide function to the 3-aminomethyl-4,6-dimethyl-2-pyrimidone moiety, known to be crucial for EZH2 inhibition (Figure 2A) [33]. The synthesis of MC3629, as well as its physical and chemical data, are reported in Supplementary Materials – Chemistry.

MC3629 was tested in the cell free enzymatic assay (EZH2/PRC2) in 10-dose IC_50_ mode with 2-fold serial dilution starting from 400 μM solution, using various histone substrates (histone H3/H4 octamer, histone H3/H4 tetramer, histone H3, and core histone) and SAM as the co-substrate. In these assays, MC3629 displayed IC_50_ values in the range 5.27-15.4 μM, with GSK2816126 used as a reference drug (IC_50_ = 9.13 nM (core histone substrate), according to the literature) [22] (Figure 2B, and Supplementary Figure 2). SAM competition experiments using histone H3/H4 octamer as the substrate confirmed its SAM-dependent mechanism of inhibition (Supplementary Figure 3). The percentages of inhibition by MC3629, used at 200 μM, towards a panel of methyltransferases including lysine (EZH1 complex, DOT1L, G9a, MLL1 complex, SET7/9), arginine (PRMT1) and DNA (DNMT1) methyltransferases definitively showed that the inhibition activity of MC3629 is selective for EZH2/PRC2 (Supplementary Table 1).

Afterwards, we tested the ability of MC3629 (at 0.5, 5, and 25 μM) to inhibit the EZH2 enzymatic activity on a cell-based assay *in vitro*. The lower significant MC3629 dosage to decrease the H3K27me3 level in MB-SLCs was 5 μM (Figure 2C).

Next, we assessed the biological effects of MC3629 on MB-SLCs by dose- and time-dependent experiments, using GSK2816126 as a reference drug. In both cell lines, we determined the lowest useful dose of MC3629 (5 μM) and the minimum effective time (48 h) (Figure 2D, 2E and Supplementary Figure 4A and 4B for data at 24 and 72 h). We next evaluated the performance of MC3629 on MB-SLCs in terms of cell viability in regards to other EZH2i in use, confirming the dose selection (Supplementary Figure 4C).

To test the specificity of the pyrazole-based EZH2 inhibitor we combined the genetic and pharmacological inhibition of EZH2 on hMB-SLCs evaluating the corresponding cell viability (Supplementary Figure 5A). In our MB-SLC the combination of MC3629 (5 μM) with shEZH2 did not display any additive effect in respect to the shEZH2 treatment alone.

To rule out nonspecific toxic effects by MC3629, we tested the EZH2i on neural stem cells (NSC) as a model of non-transformed cells [24], and we did not observe any effects on H3K27me3 and PCNA protein levels (Supplementary Figure 5B).

### Pharmacological inhibition of EZH2 impairs SHH MB cells proliferation and self-renewal, and induces apoptosis

**Figure 3:**
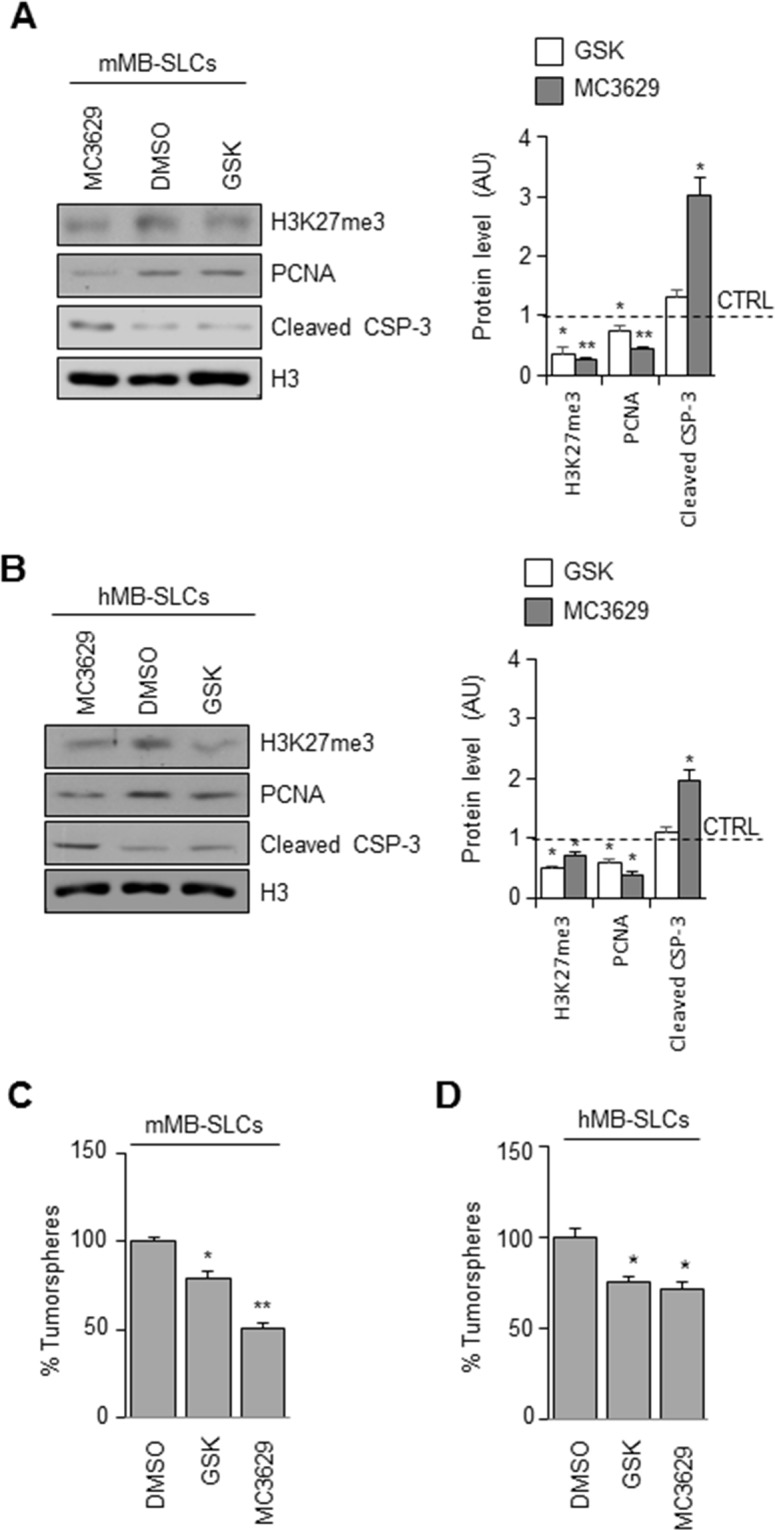
Biological effects of EZH2 inhibition in SHH MB-SLCs **(A-B)** mMB-SLCs and hMB-SLCs were treated for 48 h with 5 μM of MC3629 or GSK2816126. Representative images of Western Blot (left) and densitometric analysis (right) of H3K27me3, PCNA, and cleaved caspase 3 (cleaved CSP-3). H3: loading control. Dashed bars represent DMSO-treated cells (CTRL). *p<0.05, **p<0.01. **(C-D)** Self-renewal measured by clonogenic assay. *p<0.05, **p<0.01.

We analyzed the biological effects of MC3629 treatment in mMB-SLCs and hMB-SLCs using GSK2816126 as a reference drug. As shown in Figure 3A and 3B, both EZH2i significantly impaired H3K27me3 and PCNA protein levels, and MC3629 induced apoptosis, detected as increased level of cleaved caspase 3. Furthermore, we investigated whether the two compounds were able to impair stemness, both MC3629 and GSK2816126 significantly reduced mMB-SLC and hMB-SLC self-renewal (Figure 3C and 3D).

**Figure 4:**
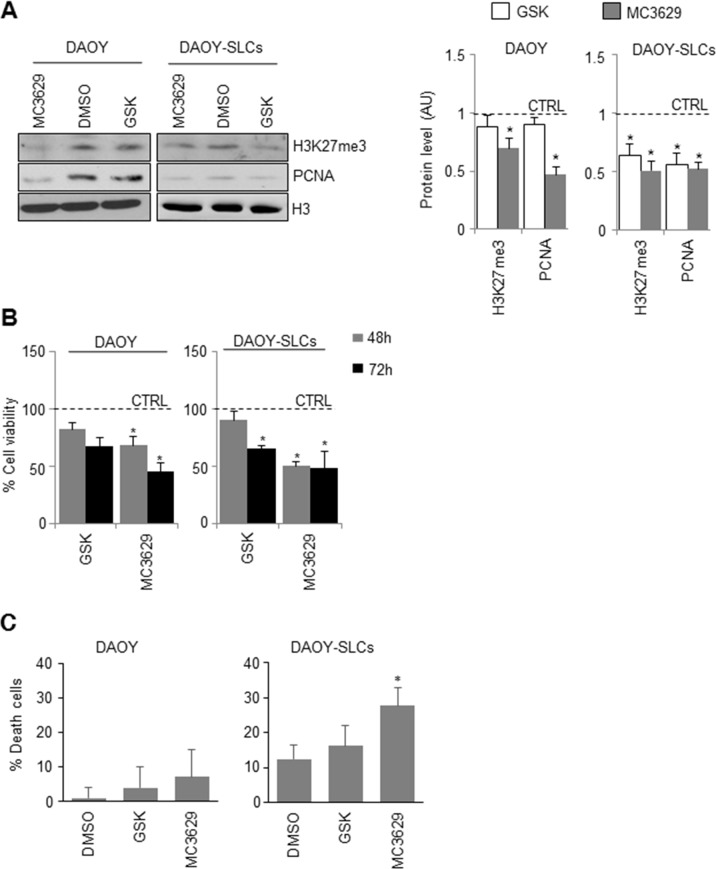
Biological effects of EZH2 inhibition in DAOY cells and DAOY-SLCs **(A)** DAOY cells and DAOY-SLCs were treated for 48 h with 5 μM of MC3629 or GSK2816126. Representative images of Western Blot (left) and densitometric analysis (right) of H3K27me3 and PCNA. H3: loading control. Dashed bars represent DMSO-treated cells (CTRL). *p<0.05. **(B)** Evaluation of cell viability, measured with MTS assay, of DAOY and DAOY-SLCs treated for 48 h and 72 h with 5 μM of MC3629 or GSK2816126. *p<0.05. **(C)** Percentage of cell death assessed in DAOY and in DAOY-SLCs treated with both compounds for 72 h. Bars represent the mean of at least three independent experiments performed in triplicate mean±S.D. *p<0.05.

We then tested the effects of MC3629 and GSK2816126 in DAOY and DAOY-SLCs. In DAOY cells, H3K27me3 and PCNA levels resulted impaired only by MC3629 (Figure 4A). In DAOY-SLCs, H3K27me3 and PCNA levels were down modulated by both drugs (Figure 4A). Such effects were confirmed in DAOY cells and DAOY-SLCs: MC3629 impaired cell viability in both cell systems after 48 h, while GSK2816126 was able to significantly impair DAOY-SLCs viability after 72 h of treatment (Figure 4B). Moreover, MC3629 induced cell death in DAOY-SLCs (Figure 4C).

Altogether, our results show that EZH2 pharmacological inhibition is able to impair MB cells proliferation and stemness, and induce apoptosis.

### MC3629 impairs SHH MB-SLCs growth and stemness *in vivo*

First, we tested both MC3629 and GSK2816126 in the parallel artificial membrane permeability assay for the BBB (PAMPA-BBB), to measure the effective permeability (Pe, cm/s) of MC3629 through a porcine brain lipid (PBL) extract impregnated on a solid filter support, used as an artificial lipid membrane [34]. Propranolol and furosemide were used as positive and negative controls, respectively. In this assay, MC3629 displayed a closer capability to permeate the lipid membrane to propranolol than GSK2816126, as highlighted by the Δ values (Table 1 and Supplementary Figure 6).

**Table 1:** Log Pe values of tested compounds in the PAMPA-BBB

COMPOUND	Log P_e_	Δ *
GSK2816126	−5.41 ± 0.04	−0.20
MC3629	−5.33 ± 0.01	−0.12
propranolol (positive control)	−5.21 ± 0.06	
furosemide (negative control)	under detection limit	

* Respect to propranolol.

**Figure 5:**
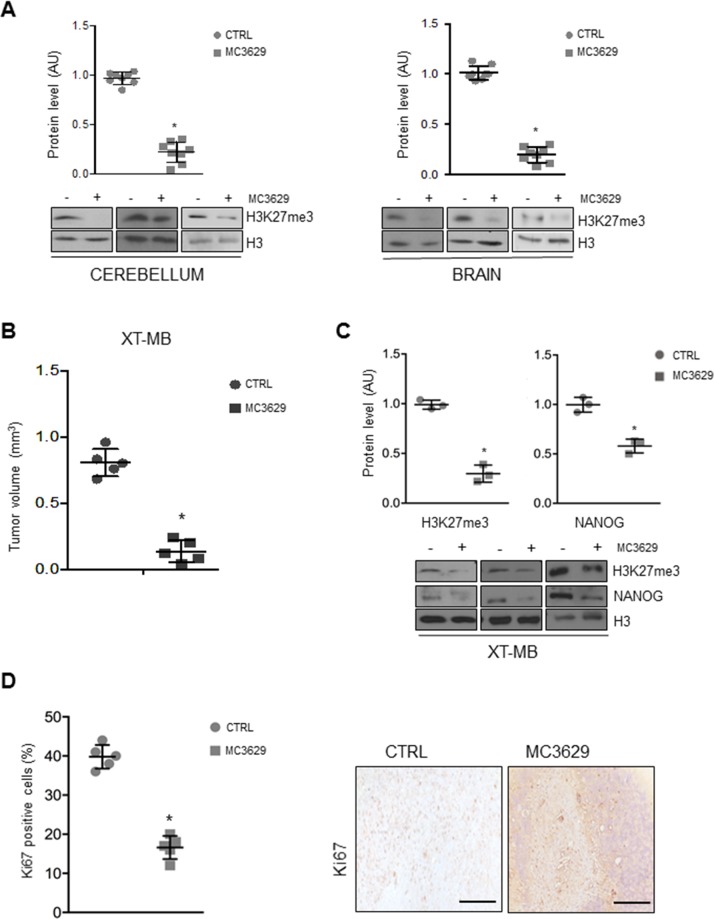
Biological effects of MC3629 *in vivo* **(A)** Representative Western Blots show H3K27me3 levels in cerebellum and brain from wild type mice treated with MC3629 or vehicle (CTRL). Dot plots represent densitometric analysis of all samples. *p<0.05. **(B-D)** Xenograft tumors (XT-MB) were generated in mice, after 10 days MC3629 was administered twice a week for 3 weeks; control mice were treated with vehicle (CTRL). (B) Xenograft volume (mm^3^) evaluated at the maximum diameter; dot plot represents mean±S.D. *p<0.05. (C) Representative images of Western Blot and densitometric analysis of H3K27me3 and NANOG. H3: loading control *p<0.05. (D) Xenograft sections were immunostained for the proliferation marker Ki67: on the left, dot plot shows Ki67 levels of proliferating cells; on the right, representative images of the staining. *p<0.05.

Dose finding experiments showed that twice/week intra-peritoneal injection of MC3629 at 20 μMol/Kg led to a significant reduction of H3K27me3 levels in the cerebella of 6-day-old mice (Supplementary Figure 7A). At this dose, we observed reduced levels of H3K27me3 not only in cerebellum but also in brain (Figure 5A), indicating that MC3629 crosses the BBB.

Next, we generated xenograft DAOY-SLCs orthotopic tumors (XT-MB) in nude mice that grew as tumor masses with the characteristics of MBs.

Mice treatment with MC3629 significantly impaired XT-MB growth (Figure 5B), and Western Blot analysis of tumor xenografts revealed a reduction of H3K27me3 in treated mice (Figure 5C). Interestingly, tumors from treated mice also showed significantly lower levels of NANOG protein (Figure 5C), and a significant reduction of proliferating Ki67-positive cells as well (Figure 5D).

Finally, we observed induction of apoptosis, detected as increased level of cleaved caspase 3 in treated mice (Supplementary Figure 7B).

Taken together, these data show that MC3629 is effective in hampering MB-SLCs growth *in vivo*.

## DISCUSSION

Epigenetic alterations have been shown to be important in cancer initiation and progression [12, 13, 35]. Among these, histone H3K9 and H3K27 methylation play a key role in cancer due to their association with gene silencing [36–39].

Based on the EZH2 role in tumorigenesis and stem cell maintenance, studies have been focused on targeting EZH2, a H3K27 methyltransferase, as a novel cancer treatment, and aimed to identify small molecules that inhibit its catalytic activity [5, 6, 40–42].

Recently, several groups reported compounds (e.g. GSK2816126, EPZ005687, EPZ6438, El1, UNC1999 and the second generation drug tazemetostat) that directly and selectively inhibit the EZH2/PRC2 enzymatic activity [17, 42, 43]. Among them, GSK2816126 is characterized by high and selective biochemical and cellular on-target potency, assessed by decrease in H3K27 trimethylation levels, has been validated in preclinical studies for treatment of lymphoma with EZH2-activating mutations [21], and is actually in phase I clinical trial for the treatment of lymphomas, solid tumors and multiple myeloma (https://clinicaltrials.gov/ct2/results?term=GSK2816126&Search=Search).

In MB cells, pharmacological inhibition of EZH2 was achieved by the use of DZNep, an inhibitor of SAH hydrolase that inhibits EZH2 through an indirect way [12]. Nevertheless, this compound displays poor EZH2 selective inhibition [44] and is not in clinical use due to its toxicity [19].

With this study, we tested the biological activity of the catalytic EZH2 inhibitor MC3629, designed and prepared by us as a simplified analog of EPZ005687 and GSK2816126, in the SHH MB cancers cells and SLCs.

In MB-SLCs derived in our laboratory from patients affected by SHH MB or from SHH MB murine model, the genetic silencing of EZH2 led to reduction of H3K27me3 levels, reduction of proliferation, and self-renewal impairment.

In the same cellular models, we tested MC3629 using GSK2816126 as a reference drug. MC3629 at 5 μM displayed significant biological activity impairing cell proliferation and self-renewal, and inducing apoptosis. When co-administered with shEZH2 to hMB-SLCs, MC3629 did not show any additive effect on cell viability, thus confirming its selective action on the methyltransferase target.

Prompted by our results, we tested MC3629 in PAMPA-BBB assay to ascertain its capability to cross BBB *in vitro*. Moreover, we observed the ability of MC3629 to reduce H3K27me3 levels in brain and cerebellum *in vivo*. Finally, we treated MB xenografted mice with MC3629, to extend its biological activity *in vivo*. By this set of experiments, we observed a significant decrease of tumor volume, a reduction of stemness and cell proliferation, and induction of apoptosis in treated mice.

Overall, in this study we validated EZH2 as an attractive target for SHH MB cancer and cancer stem cell impairment, and showed that EZH2 pharmacological inhibition exerted by MC3629 is effective in MB cells and in MB xenografted mouse models.

## MATERIALS AND METHODS

### Drug synthesis

The synthetic procedure to prepare MC3629 as well as its physical, chemical and spectral data are reported in Supplementary Materials – Chemistry.

### EZH2 assay

*Reagents*: Reaction buffer; 50 mM Tris-HCl (pH 8.0), 50 mM NaCl, 1 mM EDTA, 1 mM DTT, 1 mM PMSF, 1% DMSO. *Reaction Conditions*: EZH2: Complex of human EZH2 (GenBank Accession No. NM_004456), (amino acids 2-end) with *N*-terminal His tag, MW= 86 kDa, human EED (NM_003797) (a-a 2-end) with *N*-terminal Flag tag, MW= 51 kDa, human SUZ12 (NM_015355) (a-a 2-end) with *N*-terminal His tag, MW = 87 kDa, Human AEBP2 (NM_153207) (a-a 2-end) with *N*-terminal His tag, MW= 53 kDa, and human RbAp48 (NM_005610) (a-a 2-end) with *N*-terminal His tag, MW = 48 kDa, co-expressed in baculovirus expression system. *Substrate*: 5 μM histone H3/H4 tetramer. Methyl donor: 1 μM *S*-adenosyl-L-[methyl-^3^H]methionine (^3^H-SAM). *Enzyme*: 100 nM EZH2 complex. *Reaction Procedure*: the indicated substrate was dissolved in freshly prepared Reaction Buffer; EZH2 was delivered into the substrate solution and mixed gently. The compound was dissolved in DMSO to a stock of 20 mM and was tested in a 10-dose IC_50_ mode with 2-fold serial dilution starting at 400 μM. The compound was delivered in DMSO into the EZH2 reaction mixture by using Acoustic Technology (Echo 550, LabCyte Inc. Sunnyvale, CA) in nanoliter range, and incubated for 15 min; ^3^H-SAM was delivered into the reaction mixture to initiate the reaction; the reaction mixture was incubated for 1 h at 30°C and then delivered to filter-paper for detection; data were analyzed using Excel and GraphPad Prism 5 software for IC_50_ curve fits. For SAM competition experiments, the same procedure was used with different SAM concentration (1, 2.5. 5, and 10 μM) and H3/H4 octamer as the substrate.

### EZH1 complex, DOT1L, G9a, MLL1 complex, SET7/9, PRMT1, and DNMT1 assays

The appropriate methyltransferase (MT) substrate (0.05 mg/ml core histone for EZH1 complex, 0.05 mg/ml oligonucleosomes for DOT1L, 5 μM histone H3 (1-21) peptide for G9a, 0.05 mg/ml nucleosomes for MLL1 complex, 0.05 mg/ml core histone for SET7/9, 5 μM histone H4 for PRMT1, and 0.001 mg/ml poly(dI-dC) for DNMT1) was added in freshly prepared Reaction Buffer (50 mM Tris-HCl (pH 8.5), 5 mM MgCl_2_, 50 mM NaCl, 0.01% Brij35, 1 mM DTT, 1% DMSO). The MT enzyme was delivered into the substrate solution and the mixture was mixed gently. Afterwards, the tested compound dissolved in DMSO was delivered into the enzyme/substrate reaction mixture by using Acoustic Technology (Echo 550, LabCyte Inc. Sunnyvale, CA) in nanoliter range, and 1 μM ^3^H-SAM was also added into the reaction mixture to initiate the reaction. The reaction mixture was incubated for 1 h at 30°C and then it was delivered to filter-paper for detection. The data were analyzed using Excel and GraphPad Prism software for IC_50_ curve fits (for EZH1 complex).

### Cell culture

DAOY cells were obtained from ATCC and cultured in MEM medium (Gibco, Invitrogen), supplemented with 10% heat-inactivated fetal bovine serum, 1% sodium pyruvate, 1% non-essential amino acid solution, 1% L-glutamine and penicillin/streptomycin. Human and mouse stem-like cells (hMB-SLC and mMB-SLC) were derived, as previously described from primary human SHH-MBs and from spontaneous tumors arisen in Ptc^+/−^ mice [24]. DAOY-SLCs were obtained as described in Alimova et al., 2012 [12].

All SLCs were maintained in DMEM/F12 supplemented with 0.6% glucose, 25 mg/ml insulin, 60 mg/ml *N*-acetyl-L-cystein, 2 mg/ml heparin, 20 ng/ml EGF, 20 ng/ml bFGF (Peprotech, Rocky Hill, NJ), 1X penicillin-streptomycin and B27 supplement without vitamin A (Gibco).

For differentiation experiments, hMB-SLCs and mMB-SLC were mechanically dissociated and plated for 48 h on D-poly-lysine coated dishes in differentiation medium (DMEM/F12 with N_2_ supplement and 2 mg/ml heparin, 0,6% glucose, 60 mg/ml *N*-acetyl-L-cysteine, 1% FBS).

For self-renewal assessment, clonogenic assay was performed as previously described [24]. Briefly: oncospheres were disaggregated with non-enzymatic cell dissociation buffer (Sigma) and cells were plated at clonal density (1-2 cells/mm^2^) into 96-well plates and cultured for an appropriate number of days (7-15) until oncospheres were countable.

### Infection, treatments and cell viability assay

For lentiviral-transduction of specific anti-EZH2, short hairpins lentiviral particles were purchased from Sigma: MISSION shRNA-non-target control Transduction Particles (SCH002V); three human Lenti ShEZH2: MISSION shRNA EZH2 Lentiviral (SHCLNV) Clones TRCN0000010475, TRCN0000040074 and TRCN0000040077; three murine Lenti ShEZH2: MISSION shRNA EZH2 Lentiviral Clones TRCN0000304505, TRCN0000039041 and TRCN0000039042. Clone TRCN0000010475 for human and Clone TRCN0000304505 for mouse were used for the experiments since they demonstrated the best knock-down efficency with less off-target; cells were infected for 48 h.

Unless otherwise specified, cells were treated for 48 h with either GSK2816126 (Selleckchem) or MC3629 at 5 μM. To evaluate cell viability cells were plated at a density of 5×10^3^ cells/well in 96-well plates and were incubated with MTS solution (CellTiter 96® AQueous One Solution Promega).

### Fluorescence-activated cell sorting

Cells were sorted based on CD133 expression levels using a FACSAriaIII (BD Biosciences) equipped with a 633 nm laser and FACSDiva software (BD Biosciences version 6.1.3). Briefly, cells were immunostained with APC-conjugated anti-CD133 (Miltenyi Biotec) according to manufacturer's protocol, and were then subjected to cell sorting. Cells were gated using forward and side scatter (FSC-A and SSC-A) plot to identify live cells (average 65%), and were then detected in the APC channel for CD133 expression. Following this gate strategy, cells were sorted for expression of CD133 (APC- and APC+ cells) and subsequently checked for purity.

### Trypan blue test

Trypan blue exclusion test was used to count viable or death cells present in a cell suspension [45]. Each sample was measured in triplicate and repeated at least three times.

### Western blot

Western blots were performed according to standard procedures [46]. Briefly, protein lysates were prepared using RIPA buffer with fresh protease inhibitors. Lysates were separated on SDS polyacrylamide gels. Proteins were transferred on nitrocellulose membrane (0.45 micron) (PerkinElmer). Membranes were blocked for 1 h at room temperature in 5% nonfat dry milk and incubated overnight at 4°C with antibodies against EZH2 (4905S Cell signaling), H3K27me3 (07-449 Millipore), PCNA (2586 Cell signaling), ACTIN (I-19 sc-1616; Santa Cruz Biotechnology), GAPDH (ab9484, Abcam), cleaved CSP-3 (D-175 9661S; Cell Signaling) and H3 (ab1971, Abcam). HRP-conjugated secondary antibodies (Santa Cruz Biotechnology) were used in combination with enhanced chemiluminescence (ECL Amersham).

### RNA extraction and quantitative real-time PCR

RNA was isolated from cells using TriReagent (Ambion) and retro transcribed using the High Capacity cDNA Reverse Transcription kit (Thermo Scientific). cDNAs were analyzed using the ViiA™ 7 Real-Time PCR System (Thermo Scientific) SensiFAST™ Probe Lo-ROX (Bioline), and TaqMan gene expression assays for βIIITubulin (Hs-00801390_s1, Mm00727586_s1) and GFAP (Hs-00909233_m1, Mm01253033_m1) for human and mouse respectively. Results were evaluated using the 2-ΔΔCT method and mRNA quantification was normalized to 2 endogenous controls: ACTIN and GAPDH.

### PAMPA-BBB

Donor solution (500 μM or 250 μM) was prepared by diluting 10 or 5 mM DMSO compound stock solution using phosphate buffer (pH 7.4, 0.01 M). Filter membrane was coated with 5 μl of BBB-specific lipid solution prepared dissolving 16 mg of PBL (Avanti Lipids Polar, 141101P) in 600 μl of *n*-dodecane. Donor solution (150 μl) was added to each well of the filter plate. To each well of the acceptor plate 300 μl of solution (5% DMSO in phosphate buffer) were added. Each compound was tested in triplicate. The sandwich was incubated for 24 h at room temperature under gentle shaking. After the incubation time, the sandwich plates were separated and 250 μl of the acceptor plate were transferred to a UV quartz microtiter plate and measured by UV spectroscopy, using a Multiskan GO microplate spectrophotometer (Thermo Scientific) at 250−500 nm at step of 5 nm. Reference solutions (250 μl) were prepared diluting the sample stock solutions to the same concentration as that with no membrane barrier. The BBB permeability value Log P_e_ is determined applying the equation:

$$\log\;P_{e}=\;\log\left\{C\cdot \left(-\ln\left(1-\frac{{\left[\mathrm{drug}\right]}_{\mathrm{acceptor}}}{{\left[\mathrm{drug}\right]}_{\mathrm{equilibrium}}}\right)\right)\right\},$$

$$\text{where }C=\left(\frac{V_{D}\cdot V_{A}}{\left(V_{D}+V_{A}\right)\mathrm{area}\cdot \mathrm{time}}\right)$$

In this equation, V_A_ is the volume of the acceptor compartment (0.3 cm^3^), V_D_ is the donor volume (0.15 cm^3^), *area* is the accessible filter area (0.24 cm^2^), and *time* is the incubation time in seconds.

### Mice experiments

Mice were purchased from Charles River Laboratories and maintained in the Animal Facility at Sapienza University of Rome. All procedures were performed in accordance with the Guidelines for Animal Care and Use of the National Institutes of Health with the approval of the Ethics Committee for Animal Experimentation (Prot. N 03/2013) of Sapienza University of Rome.

### *In vivo* treatment

CD1 wild-type mice were treated intra-peritoneally with 20 μMoles/Kg of MC3629 (n=8 for each group). Treatment was performed at the age of 8 and 10 days post natal and mice were sacrificed at 12 days post natal. Brain and cerebellum tissues were collected for analysis.

### Medulloblastoma xenografts

DAOY-SLCs were stereotaxically implanted into the cerebellum of nude mice by using the following coordinates according to the atlas of Franklin and Paxinos: 6.6 mm posterior to the bregma; 1 mm lateral to the midline; and 2 mm ventral from the surface of skull of mice, under ketamine (100 mg/kg, i.p.)/xylazine (10 mg/kg, i.p.) anesthesia. Cells (2×10^5^ per 3 μl) were implanted at an infusion rate of 1 μl/min. Mice were separated into two groups. Group 1 (n=8) was intraperitoneally treated with MC3629 (20 μMoles/Kg) suspended in 10% (2-Hydroxypropyl)-β-cyclodextrin +1%DMSO (Sigma), Group 2 (n=8) was treated with 10% (2-Hydroxypropyl)-β-cyclodextrin +1%DMSO (Sigma). After 21 days of treatment, animals were sacrificed and brains were formalin fixed and paraffin embedded. Brain tumor volume calculation: Serial thick coronal sections (2 μm) starting from mesencephalon to the end of cerebellum were performed. The analysis was performed on 20 sections, sampled every 40 μm on the horizontal plan of the cerebellum, in which the tumor was identified and outlined at 2.5X magnification.

Every 40 μm of brain slice, hematoxylin and eosin (H&E) staining was performed. Tumor area of every slice was evaluated with a microscope (Axio Imager M1 microscope) equipped with a motorized stage and software Image Pro Plus 6.2. The following formula was used to calculate brain tumor volume: tumor volume = sum of (measured area for each slice x slice thickness x sampling frequency) [47].

### Immunohistochemistry

Paraffin-embedded sections were deparaffinized, and stained with either H&E or immunohistochemical stains as specified. Heat-induced epitope retrieval was performed with citrate buffer pH 6. Sections were incubated with ki67 primary antibody (*Thermo Scientific*) for 1 h. Ki67 expression was expressed as percentages of total cells with labeled nuclei.

## SUPPLEMENTARY FIGURES AND TABLE


