# Supplementary material for: The histone methyltransferase EZH2 as a druggable target in SHH medulloblastoma cancer stem cells
Source: Oncotarget. 2017 Aug 2;8(40):68557–70. doi: 10.18632/oncotarget.19782 (PMC5620277; doi:10.18632/oncotarget.19782)
Supplement: Supplementary file 1 [file oncotarget-08-68557-s001.pdf]

The histone methyltransferase EZH2 as a druggable target in SHH medulloblastoma cancer stem cells

SUPPLEMENTARY MATERIALS

Chemistry

The synthesis of MC3629 [1, 2] was performed by reaction between the 5-methyl-1-phenyl-1*H*-pyrazole-4-carboxylic acid **1** [3] and the 3-(aminomethyl)-4,6-dimethylpyridin-2(1*H*)-one hydrochloride **2** [4] in dry *N,N*-dimethylformamide in the presence of *N,N,N',N'*-tetramethyl-*O*-(benzotriazol-1-yl)uronium tetrafluoroborate (TBTU) and trimethylamine, according to Scheme 1.

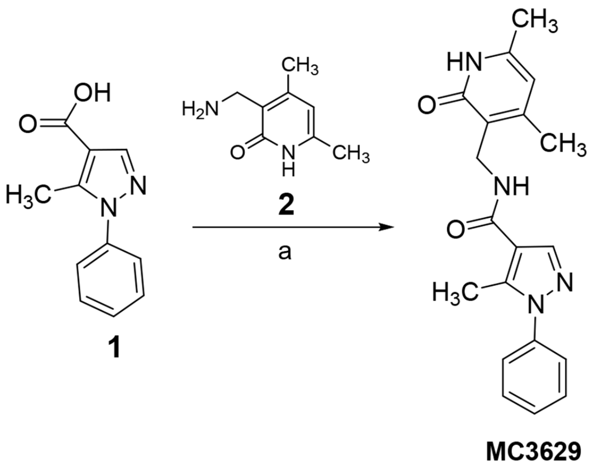

Scheme 1: Reagents and conditions: TBTU, triethylamine, dry DMF, N_2, rt, overnight.

Synthesis of *N*-((4,6-dimethyl-2-oxo-1,2-dihydropyridin-3-yl)methyl)-5-methyl-1-phenyl-1*H*-pyrazole-4-carboxamide (MC3629).

N,N,N',N'-Tetramethyl-*O*-(benzotriazol-1-yl) uronium tetrafluoroborate (TBTU) (0.89 mmol, 0.463 g) and triethylamine (5.19 mmol, 0.72 mL) were added under nitrogen flow to a solution of 5-methyl-1-phenyl-1*H*-pyrazole-4-carboxylic acid 1 [3] (0.74 mmol, 0.15 g) in anhydrous *N,N*-dimethylformamide (5 mL), and the solution was left under stirring at room temperature for 25 min. After this time, 3-(aminomethyl)-4,6-dimethylpyridin-2(1*H*)-one hydrochloride **2** [4] (0.89 mmol, 0.17 g) was added, and the mixture was stirred at room temperature overnight. The reaction was quenched by sodium chloride saturated solution (20 mL) and the precipitate was filtered off and washed with water (3 × 3 mL) and *n*-hexane (3 × 3 mL). The resulting light yellow solid was purified by recrystallization (acetonitrile:methanol 1:1) to provide pure MC3629 as a colourless solid.

Yield 65%; melting point: 225-227 °C, recrystallization solvent: methanol.

^{1}H NMR (DMSO-d_6, 400 MHz, δ, ppm): δ 2.13 (s, 3H, 4,6-dimethylpyridone CH_3), 2.19 (s, 3H, 4,6-dimethylpyridone CH_3), 2.51 (s, 3H, 5-methylpyrazole CH_3), 4.27 (d, 2H, CH_2NH, J = 4.8 Hz), 5.87 (s, 1H, CH pyridone proton), 7.53 (m, 5H, aromatic protons), 7.96 (t, 1H, CH_2NH, J = 4.8 Hz), 8.13 (s, 1H, pyrazole proton), 11.48 (br s, 1H, pyridone NH) ppm.

^{13}C NMR (DMSO-d_6, 100 MHz, δ, ppm): δ 11.1, 18.5, 19.1, 32.8, 111.1, 116.0, 123.3 (2C), 126.9, 127.2, 129.1 (2C), 139.2, 142.6, 146.6, 151.5, 158.0, 163.6, 165.9 ppm; MS (EI) calc: m/z: 336.1586 $[M]^+$ found: m/z: 336.1590 $[M]^+$.

REFERENCES

1. Albrecht BK, Audia JE, Cook AS, Dakin LA, Duplessis M, Gehling VS, Harmange JC, Naveschuk CG, Vaswani RG. Preparation of Heteroaryl Amides as Modulators of Methyl Modifying Enzymes. Google Patents. 2013; WO2013120104A2.
2. Blackledge CW Jr, Burgess JL, Johnson NW, Kasparec J, Knight SD, Lafrance LV 3rd, Luengo JI, Miller WH, Newlander KA, Romeril SP, Schulz M, Su DS, Tian X. Preparation of N-[(4,6-dimethyl-2-oxo-1,2-dihydropyridin-3-yl)methyl] amides as enhancers of zeste homolog 2 (EZH2) inhibitors. PCT Int. Appl. 2014; WO 2014177982 A1 20141106.
3. Menozzi G, Mosti L, Schenone P. Reaction of 2-Dimethylaminomethylene-1,3-Diones with Dinucleophiles. Vi. Synthesis of Ethyl or Methyl 1,5-Disubstituted 1h-Pyrazole-4-Carboxylates. J Heterocycl Chem. 1987; 24:1669–75.
4. Verma SK, Tian X, LaFrance LV, Duquenne C, Suarez DP, Newlander KA, Romeril SP, Burgess JL, Grant SW, Brackley JA, Graves AP, Scherzer DA, Shu A, et al. Identification of Potent, Selective, Cell-Active Inhibitors of the Histone Lysine Methyltransferase EZH2. ACS Med Chem Lett. 2012; 3:1091-6.

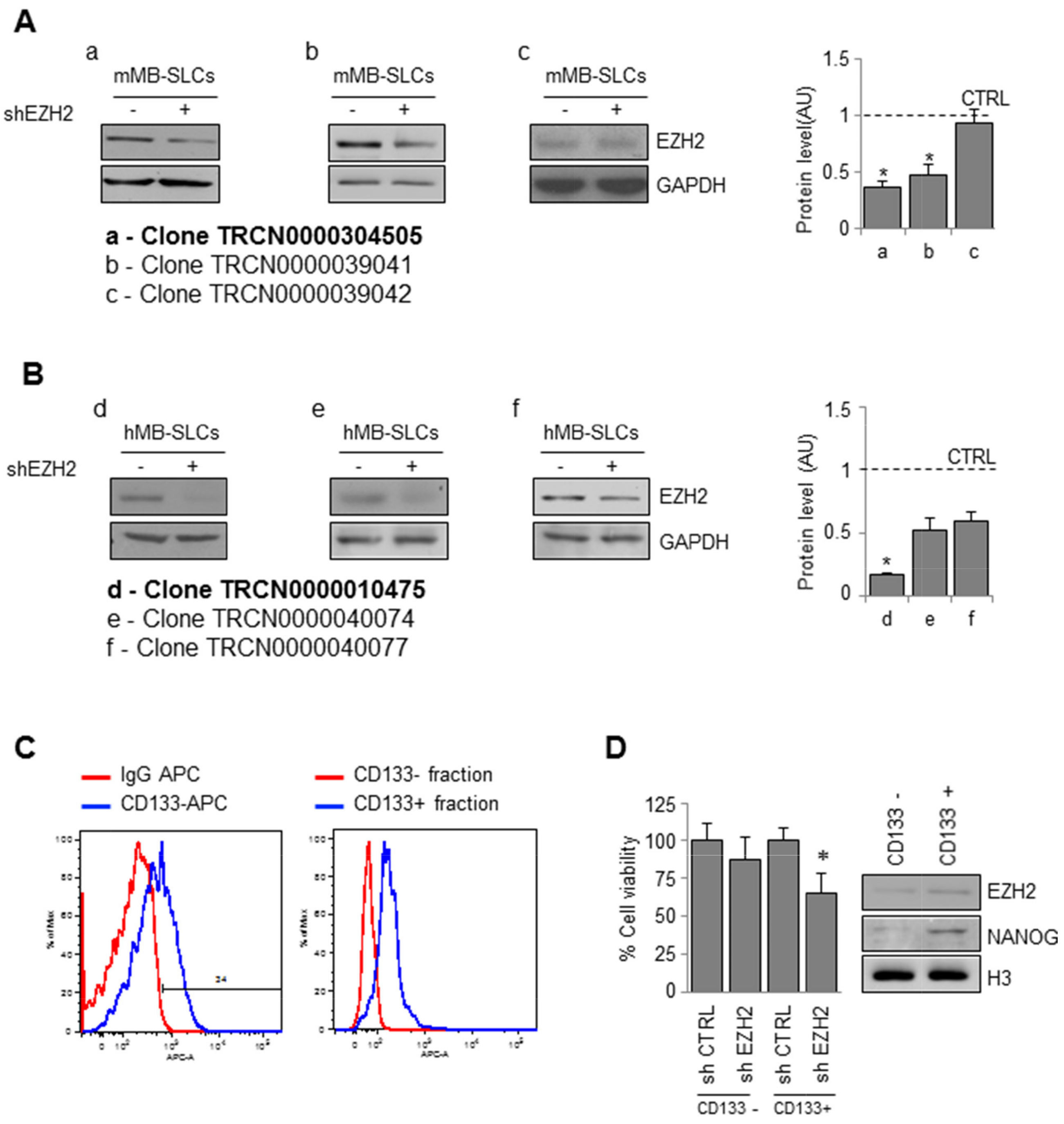

Supplementary Figure 1: (A-B) Representative images of Western Blot (left) and densitometric analysis (right) for EZH2 before and after shEZH2 in mMB-SLCs and hMB-SLCs; GAPDH: loading control *p<0.05. (A) Images represent the efficiency of three different clones for mouse Lenti shEZH2: TRCN0000304505, TRCN0000039041 and TRCN0000039042. (B) Images represent the efficiency of three different clones for human Lenti shEZH2(SHCLNV): TRCN0000010475, TRCN0000040074 and TRCN0000040077. **(C)** hMB-SLCs were analyzed for CD133 expression resulting in 34% of positive cells and sorted to have enriched CD133 positive and negative fractions (left). **(D)** Cell count (left) of viable CD133 sorted hMB-SLCs after and before shEZH2 *p<0.05. Representative western Blot (right) for EZH2, NANOG in CD133 positive and negative fractions. H3: loading control.

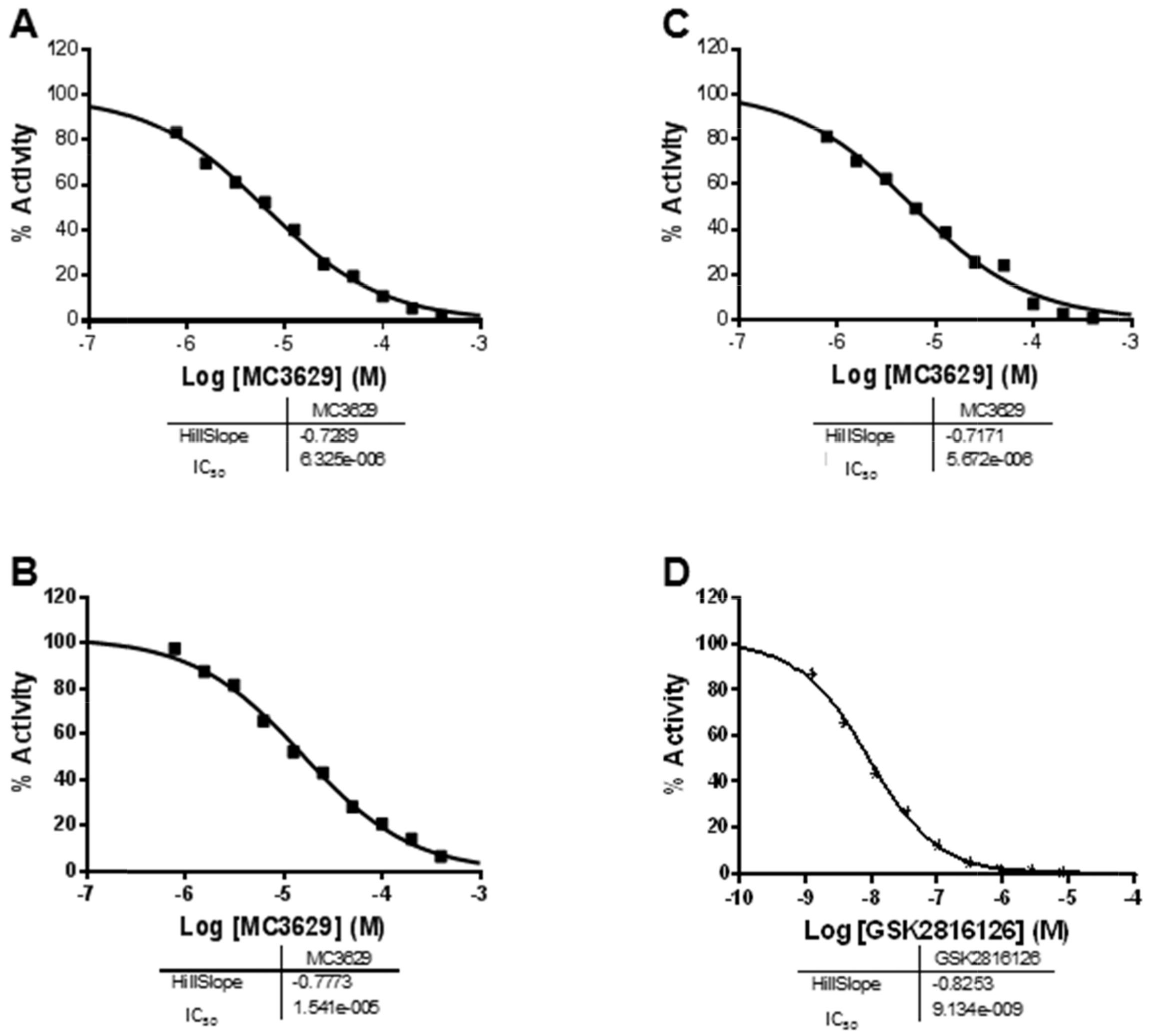

Supplementary Figure 2: Dose-response activities and IC_{50} values of EZH2/PCR2 complex with MC3629 in 10-dose IC_{50} mode with 2-fold serial dilution starting from 400 μM compound solution in DMSO using histone H3/histone H4 octamer **(A)**, histone H3 **(B)**, and core histone **(C)** substrates. SAM was used as the co-substrate. The dose-response curve and relative IC_{50} for GSK2816126 (core histone substrate) **(D)** is also shown.

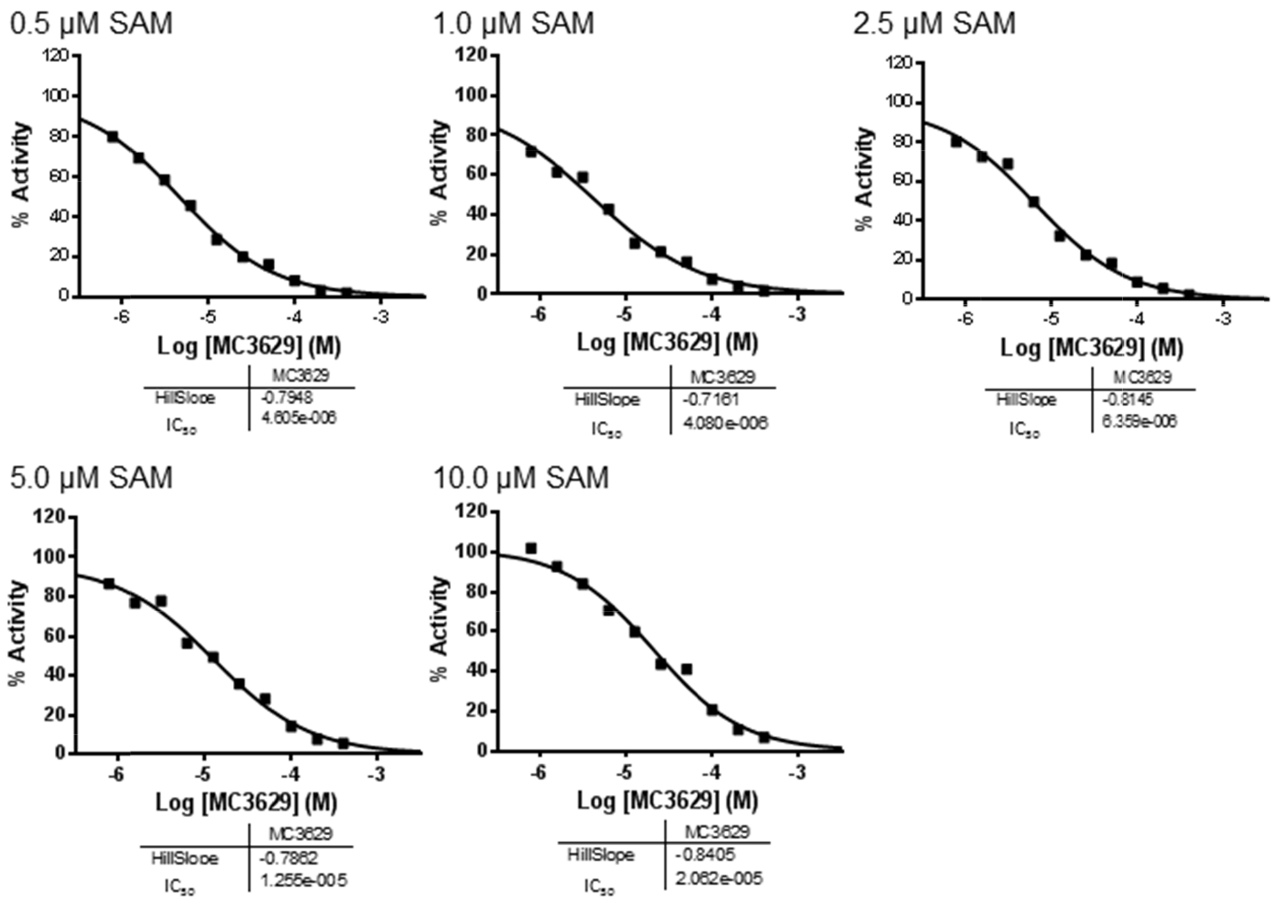

Supplementary Figure 3: SAM-dependence of the EZH2/PRC2 complex inhibition by MC3629 with increasing SAM concentration.

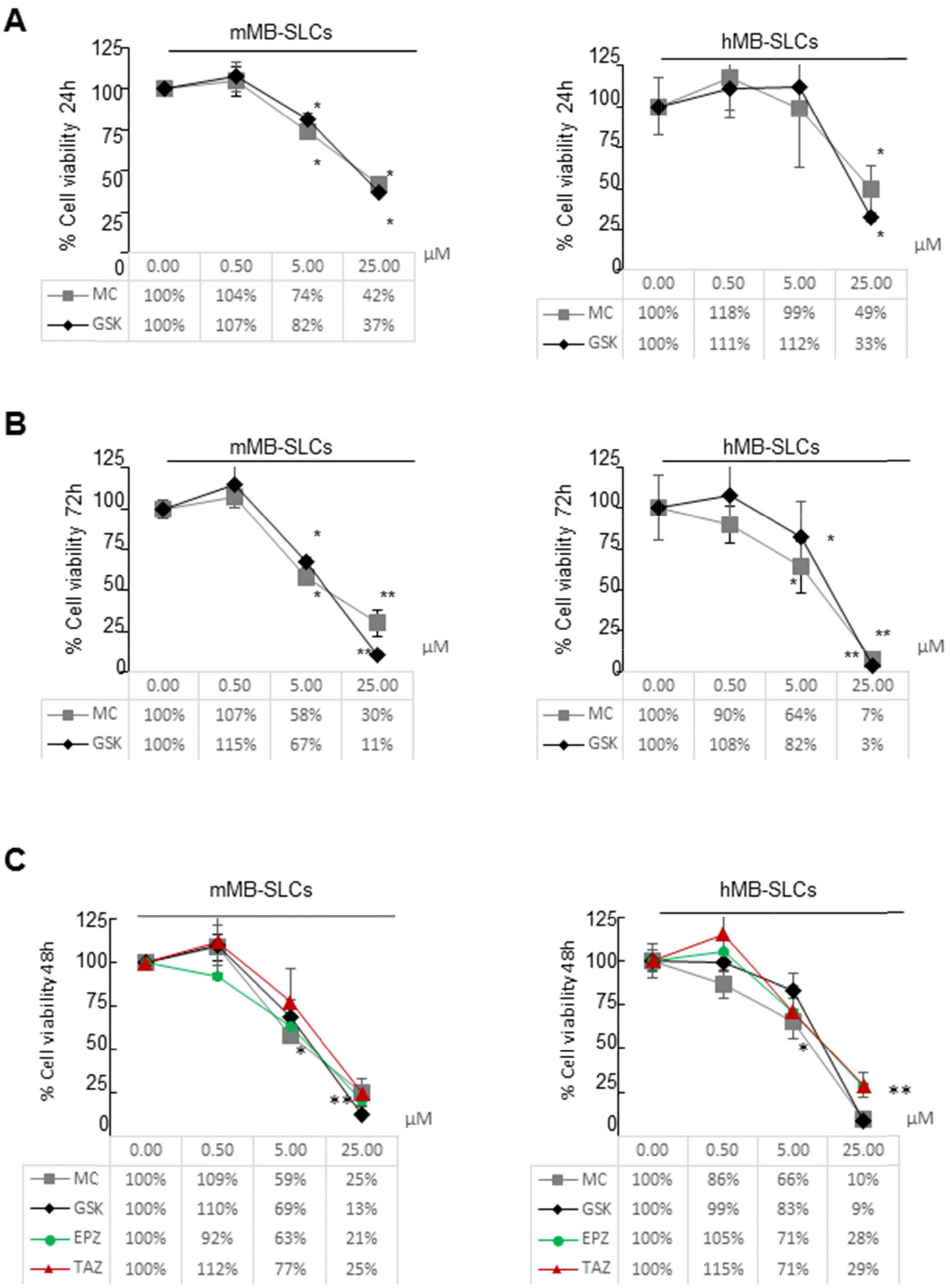

Supplementary Figure 4: (A-C) Evaluation of cell viability, measured with MTS assay, of mMB-SLCs and hMB-SLCs treated for 24 h (A), 72 h (B) with 0.5, 5, 25 µM of MC3629 or GSK2816126 and (C) 48 h with 0.5, 5, 25 µM of MC3629, GSK2816126, EPZ005687 and tazemetostat. *p<0.05, **p<0.01.

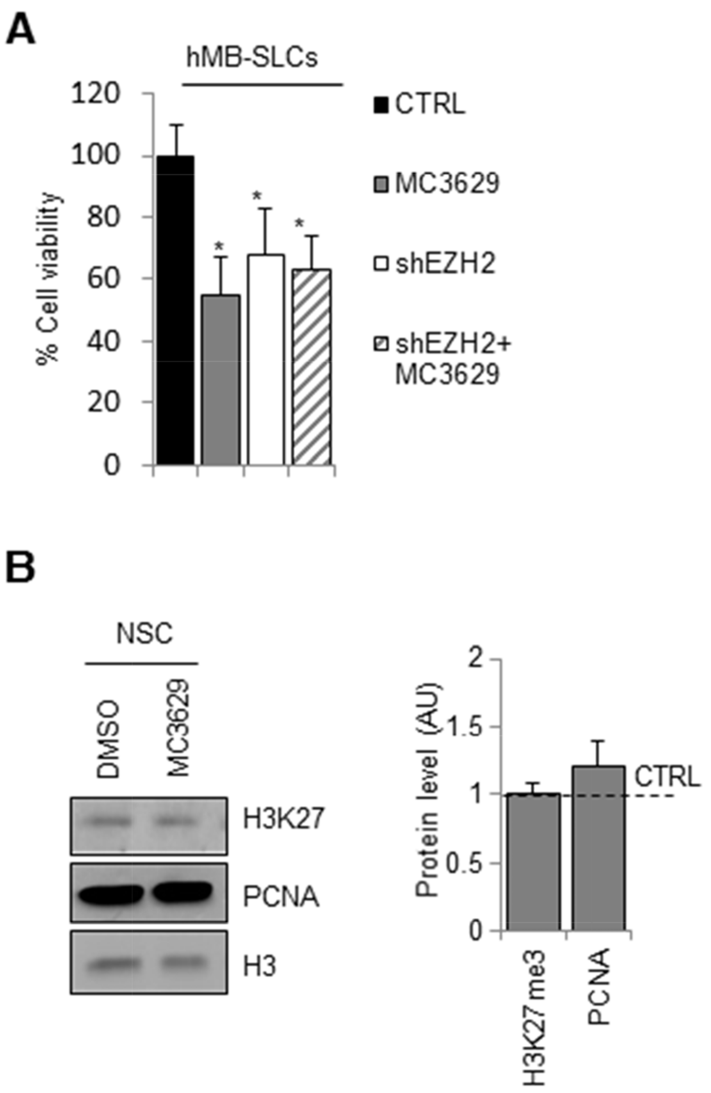

Supplementary Figure 5: (A) Evaluation of cell viability, measured with MTS assay, of hMB-SLCs treated for 48 h with 5 µM of MC3629 and shEZH2 alone or in combination. Bars represent mean±S.D. *p<0.05. **(B)** Western Blot (left) and densitometric analysis (right) of H3K27me3 and PCNA, in Neural Stem Cells (NSC) treated for 48 h with 5 µM MC3629. H3: loading control. *p<0.05.

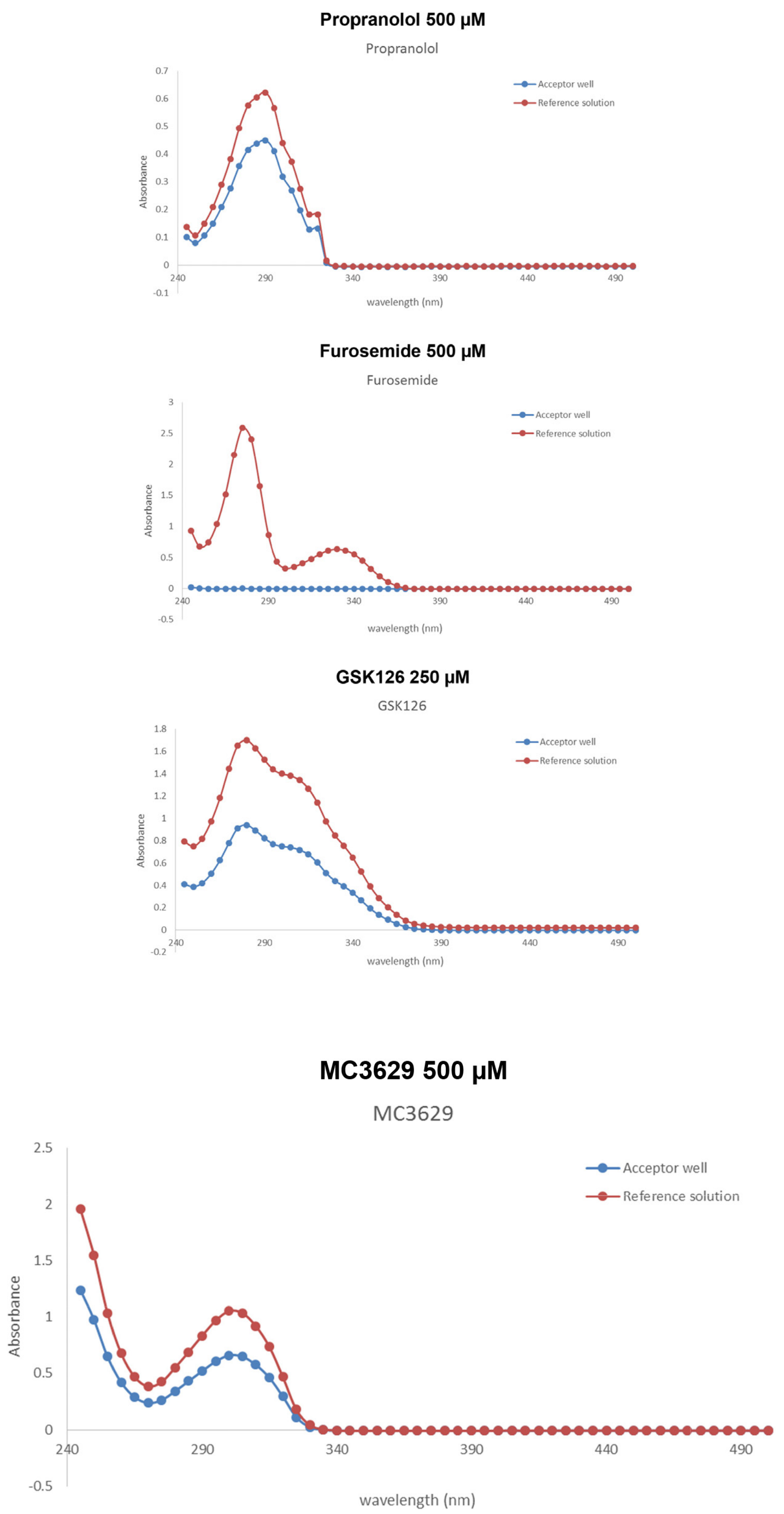

Supplementary Figure 6: PAMPA-BBB assay. Absorbance values for the specific compound [propranolol 500 µM (positive control), furosemide 500 µM (negative control), GSK126 250 µM (lower concentration due to solubility problems), and MC3629 500 µM] in the reference solution (red) and in the acceptor well (blue).

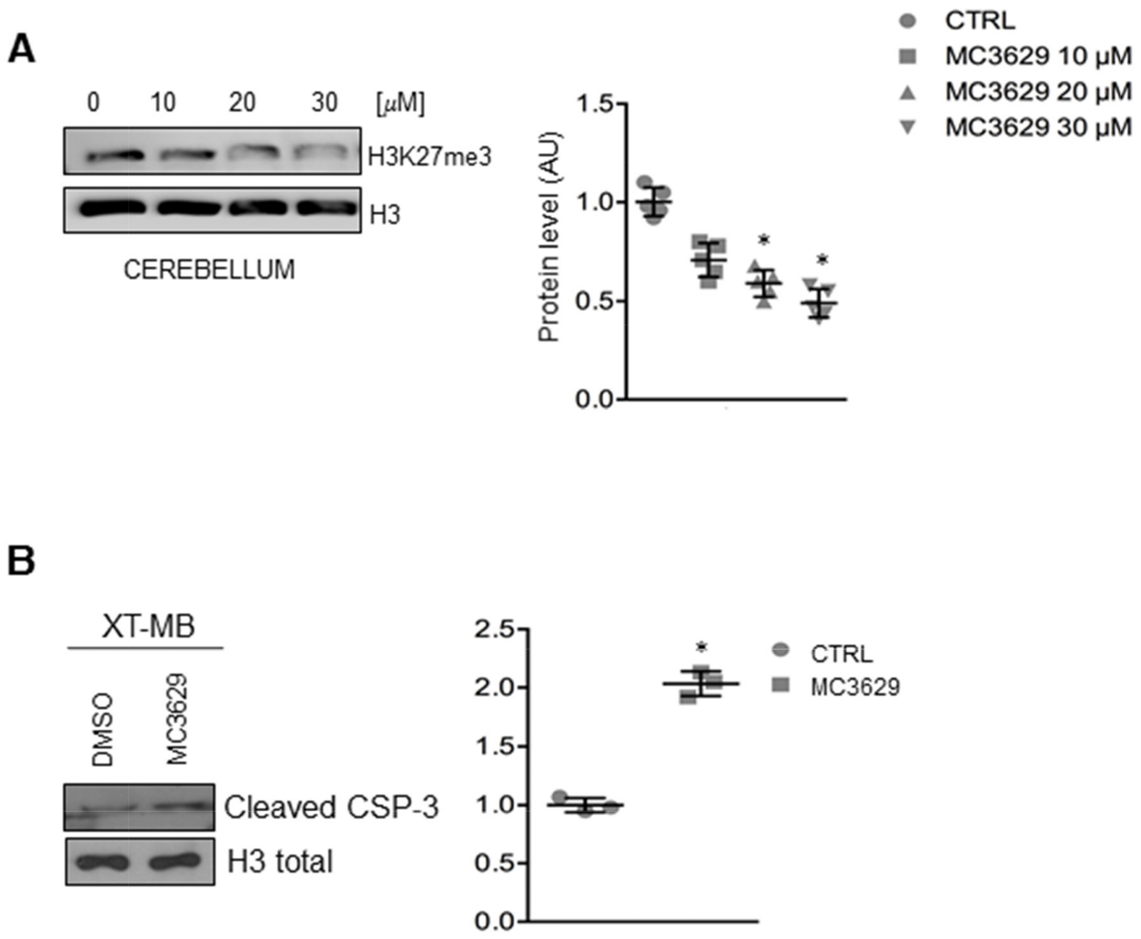

Supplementary Figure 7: (A) Representative western Blot (left) and densitometric analysis (right) of H3K27me3 for dose finding in *in vivo* experiments. H3: loading control, *p<0.05. **(B)** Representative Western Blot (left) and densitometric analysis (right) of cleaved-CSP-3 for evaluation of apoptosis in XT-MB treated with MC3629. H3: loading control. *p<0.05.

Supplementary Table 1: Percentage of inhibition of MC3629 (200 µM) against a panel of methyltransferases

MT	substrate	% inhibition
EZH1 complex	histone H3/H4 octamer	62.4*
DOT1L	nucleosomes	3
G9a	histone H3 1-21	5.1
MLL1 complex	nucleosomes	20.7
SET7/9	histone H3/H4 octamer	5.2
PRMT1	histone H4	5.7
DNMT1	Poly dl-dC	0

*IC_{50} = 138.5 µM.
